# The Association of Fasting C-peptide with Corneal Neuropathy in Patients with Type 2 Diabetes

**DOI:** 10.1155/2020/8883736

**Published:** 2020-12-02

**Authors:** Anju Zuo, Chuan Wang, Lili Li, Jingru Qu, Juan Cao, Li Chen, Solomon Tesfaye, Wenjuan Li, Xinguo Hou

**Affiliations:** ^1^Department of Endocrinology, Qilu Hospital of Shandong University, Jinan, Shandong, China 250012; ^2^Department of General Practice, Qilu Hospital of Shandong University, Jinan, Shandong, China 250012; ^3^Institute of Endocrine and Metabolic Diseases of Shandong University, Jinan, Shandong, China 250012; ^4^Key Laboratory of Endocrine and Metabolic Diseases, Shandong Province Medicine & Health, Jinan, Shandong, China 250012; ^5^Jinan Clinical Research Center for Endocrine and Metabolic Diseases, Shandong Province Medicine & Health, Jinan, Shandong, China 250012; ^6^Department of Ultrasound, Qilu Hospital of Shandong University, Qingdao, Shandong, China 266000; ^7^Department of Health Management Center, Qilu Hospital of Shandong University, Jinan, Shandong, China 250012; ^8^Diabetes Research Department, Sheffield Teaching Hospitals NHS Foundation Trust, Sheffield, UK

## Abstract

**Purpose:**

Damage to corneal nerve fibers has been demonstrated in people with type 2 diabetes mellitus (T2DM) that further progresses with increasing severity of diabetic peripheral neuropathy. However, the role of C-peptide in corneal nerve damage has not been reported in T2DM. The present study investigated the relationship of fasting C-peptide levels with corneal neuropathy evaluated by corneal confocal microscopy (CCM) in patients with T2DM.

**Methods:**

160 T2DM patients (72 females) aged 34-78 with duration ranging from 0 to 40 years underwent CCM to measure corneal nerve fiber length (CNFL), corneal nerve fiber density (CNFD), and corneal nerve branch density (CNBD). Pearson correlation analysis and multiple linear regression analysis were used to explore the association of fasting C-peptide levels with corneal nerve parameters. Partial correlation analysis (adjusted for age and gender) was also conducted to analyze the correlation of metabolic indexes with these three corneal nerve parameters. The relationship between fasting C-peptide levels and duration of diabetes was also explored by Pearson correlation analysis.

**Results:**

With an increase in fasting C-peptide levels, the values of CNFL, CNFD, and CNBD also showed a corresponding trend for an increase. Partial correlation analysis revealed that fasting C-peptide levels were positively associated with CNFL (*r* = 0.245, *P* = 0.002), CNFD (*r* = 0.180, *P* = 0.024), and CNBD (*r* = 0.214, *P* = 0.008) after adjusting for age and gender. Using multiple linear regression analysis, fasting C-peptide levels were also closely associated with CNFL (*P* = 0.047) and CNBD (*P* = 0.038) after multiple adjustments. However, this association disappeared after further adjusting for duration of diabetes. Further analysis indicated that fasting C-peptide levels declined with duration of diabetes (*r* = −0.267, *P* = 0.001).

**Conclusions:**

C-peptide was closely associated with corneal neuropathy and disease duration in T2DM. C-peptide levels might be both an indicator of beta-cell function and a marker of disease severity (such as diabetic corneal neuropathy) and duration.

## 1. Introduction

The prevalence of diabetic peripheral neuropathy (DPN) was estimated 8-45% in patients with type 2 diabetes mellitus (T2DM) [1]. DPN could lead to distressing neuropathic pain, insensitivity to trauma leading to foot ulceration, and autonomic neuropathy affecting several systems, all resulting in significant morbidity and increased mortality [2, 3]. Injury of the small nerve fibers is probably the earliest manifestation of DPN [4, 5]. However, DPN is often diagnosed based on neurological and electrophysiological testing of large nerve fibers in clinical practice, which results in delayed diagnosis [6]. At present, apart from tight glycemic control in T1DM, there are no effective treatments that can reverse or prevent the progression of DPN [7]. Many compounds that showed promising results in animal models of DPN have not translated to human clinical trials. This has in part been due to the lack of sensitive early markers/measures of DPN. Therefore, the development of a reliable method of detecting early damage of small nerve fibers and the search for potential protective factors of these small nerve fibers would be of great value.

Corneal confocal microscopy (CCM) is an objective and noninvasive imaging technique that has been validated to quantify corneal small nerve fibers [8]. Corneal nerve loss has been found in recently diagnosed people with T2DM [9] and is strongly associated with the severity of DPN [10]. Moreover, corneal nerve regeneration on CCM has been demonstrated in T1DM patients following simultaneous pancreas and kidney transplantation (SPK) [11, 12]. Additionally, the predictive validity of CCM for DPN has been reported in a prospective study, with a baseline corneal nerve fiber length (CNFL) of <14.9 mm/mm^2^ being the strongest predictor [13]. Therefore, CCM is not only a powerful diagnostic tool for the early detection of DPN but also an effective evaluation parameter for the therapeutic effect of interventions in DPN.

The protective effect of C-peptide on DPN in T1DM had been confirmed by various studies [14–17]. However, the relationship between C-peptide and DPN in T2DM remains unclear. Several studies have found that C-peptide had a protective effect on DPN [18–21], while others have not observed such an effect [22] or indeed observed a contrary result [23]. To our knowledge, no study investigated the relationship of C-peptide levels with corneal nerve loss/damage in T2DM.

The present study investigated the relationship of fasting C-peptide levels with corneal neuropathy evaluated by CCM in patients with T2DM.

## 2. Methods

### 2.1. Study Population

We recruited 160 T2DM patients (72 females) aged 34-78 with duration ranging from 0 to 40 years at Qilu Hospital of Shandong University from March 2016 to December 2019. Exclusion criteria were as follows: history of (1) acute myocardial infarction, acute cerebral infarction, cerebral hemorrhage, tumor, infectious disease, severe hepatic, or renal disease; (2) corneal trauma, ocular disease, or systemic disease that might affect the cornea; (3) cigarette smoking and excessive alcohol consumption; and (4) taking any of the following medications: insulin secretagogues and statins. Diabetes was diagnosed based on the 2006 World Health Organization (WHO) criteria [24]. The study protocol adhered to the provisions of the Helsinki Declaration and was approved by the ethics committee of Qilu Hospital. All subjects signed informed consent to participate in the study.

### 2.2. Data Collection

Basic demographic information was collected by the data collection system of Qilu Hospital and structured history. These included age, gender, BMI, blood pressure (BP), duration of diabetes, history of smoking and alcohol consumption, previous medical history, and medication history. Antidiabetic medications were classified as follows: insulin, metformin, alpha glucosidase inhibitor, and others (thiazolidinedione, dipeptidyl peptidase-4 inhibitors, and others). After fasting for at least 10 hours, venous blood was collected for the measurement of glucose, HbA1c, triglycerides, total cholesterol (TC), and creatinine by use of an automatic analyzer (ARCHITECT ci16200 Integrated System, Abbott, USA). Plasma C-peptide levels were measured at the hospital laboratory using the Cobas e 602 analyzer (Roche Diagnostics, Mannheim, Germany). The calibration of the assay ranged from 0.06 to 6.2 ng/mL. Patients on long-acting insulin treatment stopped insulin injections 24 hours before blood collection. All subjects underwent CCM examination using the Heidelberg Retina Tomograph Rostock Cornea Module (HRT-III, Heidelberg, Germany) according to a standard procedure [12]. Three, nonoverlapping images from the center of the cornea were selected for quantification from each eye of each person. Data acquired from these images were averaged for each eye, and the mean values of both eyes were used for analysis. Three corneal nerve parameters were measured: CNFL was calculated in mm/mm^2^ as the total length of all nerve fibers and branches; CNFD indicated the total number of major nerves per mm^2^; CNBD indicated the number of branches emanating from the major nerve trunks per mm^2^.

### 2.3. Statistical Analysis

**Figure 1:**
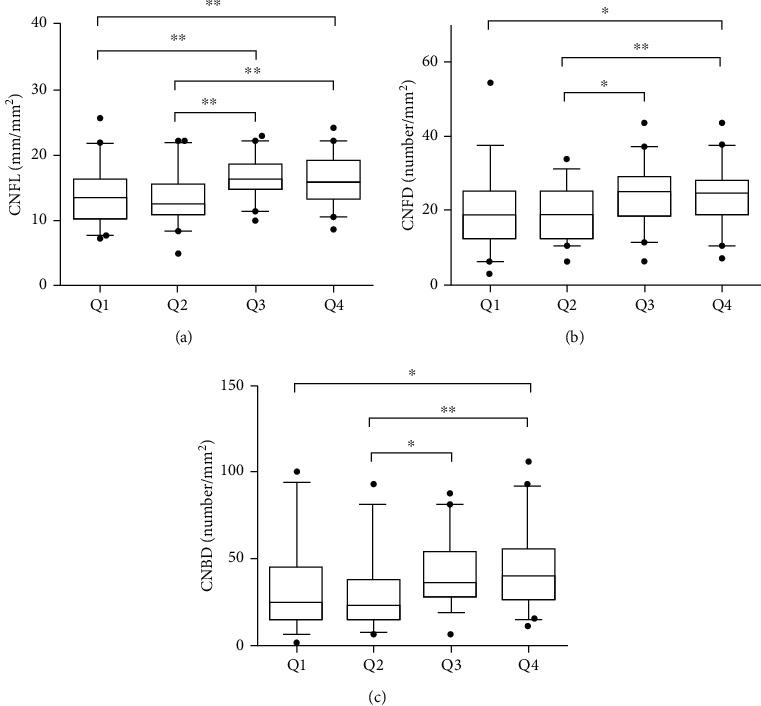
Differences of CNFL, CNFD, and CNBD between groups based on the quartiles of fasting C-peptide levels. ^∗^*P* < 0.05 and ^∗∗^*P* < 0.01.

Statistical analyses were performed using SPSS 22.0 software (SPSS Inc., Chicago, USA). Firstly, the data were assessed for normality using the Kolmogorov-Smirnov test. Data were expressed as the means ± SD or median (interquartile range) or number (%). The differences between groups were compared by one-way ANOVA (LSD) (continuous data with normal distribution) or Mann–Whitney *U* test (continuous data with skewed distribution) or chi-square test (categorical data). The values of the three corneal nerve parameters were expressed as box and whiskers with 5-95 percentiles in Figure 1. Pearson correlation analysis and multiple linear regression analysis were used to explore the association of fasting C-peptide levels with three corneal nerve parameters. Partial correlation analysis (adjusted for age and gender) was conducted to analyze the correlation of metabolic indexes with three corneal nerve parameters. The relationship between fasting C-peptide levels and duration of diabetes was also explored by Pearson correlation analysis. *P* < 0.05 was considered statistically significant.

## 3. Results

### 3.1. General Characteristics of Participants

All subjects were divided into four groups based on the quartiles of fasting C-peptide levels. As shown in Table 1, with increasing fasting C-peptide levels, BMI also increased. Patients with the lowest fasting C-peptide levels presented the highest TC levels. Moreover, patients with higher fasting C-peptide levels had a shorter duration of diabetes. The choice of hypoglycemic treatment was also different between groups. We then compared the differences of CNFL, CNFD, and CNBD between groups (Table 1 and Figure 1). Overall, with the increase of fasting C-peptide levels, the values of CNFL, CNFD, and CNBD also showed an increasing trend.

### 3.2. The Correlation of Fasting C-peptide Levels and Metabolic Indexes with Corneal Nerve Parameters

**Figure 2:**
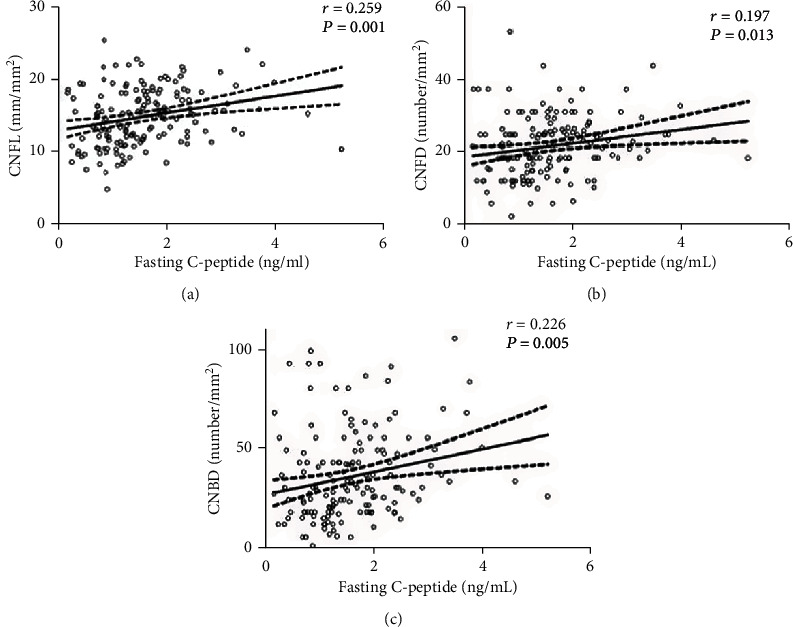
Correlation of fasting C-peptide levels with CNFL, CNFD, and CNBD.

In order to assess the correlation of fasting C-peptide levels with corneal nerve parameters, we firstly conducted a Pearson correlation analysis and observed that fasting C-peptide levels were positively associated with CNFL, CNFD, and CNBD (Figure 2). Then, we carried out a partial correlation analysis to further explore the correlation of fasting C-peptide levels and other metabolic indexes with corneal nerve parameters. As shown in Table 2, after adjusting for age and gender, fasting C-peptide levels were also positively associated with CNFL, CNFD, and CNBD. Moreover, a negative correlation of HbA1c, duration of diabetes, and TC with CNFL, but not with CNFD and CNBD, was observed.

### 3.3. Multiple Linear Regression Analysis of Fasting C-peptide Levels with Corneal Nerve Parameters

**Figure 3:**
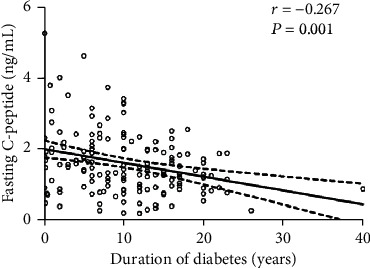
Correlation of duration of diabetes with fasting C-peptide levels.

Multiple linear regression analysis was then used to further investigate the correlation of fasting C-peptide levels with corneal nerve parameters in different models. As shown in Table 3, before adjustment, fasting C-peptide levels were closely associated with CNFL, CNFD, and CNBD. Then, we added age, gender, BMI, SBP, HbA1c, triglyceride, TC, eGFR, insulin treatment, metformin, alpha glucosidase inhibitor, and other antidiabetic medications into the model. Fasting C-peptide levels were also closely associated with CNFL and CNBD. As DPN was closely tied to the duration of disease, we further put duration of diabetes into the model (model 3). Unfortunately, fasting C-peptide levels no longer showed a close association with corneal nerve parameters. Finally, we assessed the relationship between fasting C-peptide levels and duration of diabetes. As shown in Figure 3, fasting C-peptide levels declined with duration of diabetes.

## 4. Discussion

DPN was one of the most common microvascular complications in diabetes. DPN could affect both large and small nerve fibers, and small nerve fiber damage was an earlier parameter of DPN [4, 5]. Therefore, the search for an objective and sensitive assessment method for small nerve fiber damage is very important for the early diagnosis and prevention of DPN. Skin biopsy is a well-established method to evaluate small nerve fiber damage [25]. However, its invasive feature limits its routine use in clinical practice [26]. Recently, CCM, an objective and noninvasive imaging technique, has been developed as an alternate measure of DPN and has been discussed in detail in several recent reviews [27, 28]. Corneal nerve assessment could be used not only for the early diagnosis of DPN but also for the evaluation of the efficacy of an intervention. There is now a wealth of studies that confirm the utility of corneal parameters including CNFL, CNFD, and CNBD that are able to distinguish patients with and without DPN [27].

At present, there are no universally accepted disease-modifying treatments that can reverse or prevent progression of DPN [7]. Therefore, the identification of potential protective factors for DPN would be very useful in tackling the disease. The protective role of C-peptide for DPN in T1DM has been well recognized [14–17]. However, this benefit remains unclear in T2DM. Recently, Zaharia et al. [29] confirmed an association between a phenotype of severe insulin-deficient diabetes (not autoimmune) and diabetic sensorimotor polyneuropathy in the newly diagnosed population of the German Diabetes Study, indicating the possible protective role of C-peptide (or insulin) on DPN. Actually, most studies conducted in T2DM suggested a protective effect of C-peptide on DPN [18–21]. However, Sari and Balci [22] demonstrated that there was no relationship between C-peptide and sensory neuropathy. Gottsäter et al. [23] even found that patients with parasympathetic neuropathy presented with higher fasting C-peptide levels. The conflicting results might be partly ascribed to the different assessment methods of DPN in these studies. Therefore, the relationship between C-peptide and DPN in carefully designed studies may provide much needed clarification.

Since CCM had been widely used as a sensitive measure of DPN and no study had been done to explore the association of C-peptide with corneal nerve damage in patients with T2DM, we conducted this cross-sectional study. We found that with an increase in fasting C-peptide levels, CNFL, CNFD, and CNBD also showed a corresponding increase. Both Pearson correlation analysis and partial correlation analysis showed that fasting C-peptide levels were positively associated with CNFL, CNFD, and CNBD. Even after adjusting for age, gender, BMI, SBP, HbA1c, triglyceride, TC, eGFR, insulin treatment, metformin, alpha glucosidase inhibitor, and other antidiabetic medications in a multiple linear regression analysis, this positive correlation also persisted. All these results indicate a possible protective effect of fasting C-peptide on corneal nerves in T2DM. The molecular mechanisms involved in the protective effect of C-peptide on neuropathy mainly come from animal studies in T1DM [30]. In BB/Wor rats, C-peptide administration prevented the development of nerve conduction velocity (NCV) deficits [31]. Further study found that administration of C-peptide in physiological concentrations corrected the endoneurial perfusion deficit caused by diabetes via a NO-sensitive mechanism [32]. Moreover, C-peptide in physiological concentrations could partially correct the reduction of Na^+^-, K^+^-ATPase activity induced by diabetes thereby reducing the observed paranodal swelling through reduced Na^+^ retention [31, 33].

Corneal nerve damage is not only associated with DPN but is also an important risk factor for diabetic neurotrophic keratopathy, a manifestation of ocular surface dysfunction [34]. Corneal nerves provide trophic support to the corneal epithelial cells by secreting mediators and epithelial cells, and these in turn provide trophic support to corneal neurons by releasing growth factors [35]. However, diabetes reduces the concentrations of these mediators, leading to damage of epithelial integrity and further neuronal loss, eventually resulting in diabetic neurotrophic keratopathy [36]. We found the positive association of C-peptide with corneal nerve parameters in T2DM patients. The role of C-peptide in diabetic neurotrophic keratopathy remains to be further studied. As DPN was closely tied to increasing duration of diabetes [37] and hence decline in *β*-cell function, there is a clear rationale to study the relationships between diabetes duration, C-peptide, and the extent of corneal nerve damage. First, the partial correlation analysis revealed that duration of diabetes is negatively associated with CNFL, which is consistent with previous studies [38]. Then, we put duration of diabetes into the model of multiple linear regression analysis to see the relationship between fasting C-peptide levels and corneal nerve parameters. However, a clear relationship between fasting C-peptide levels and corneal nerve parameters was no longer observed, indicating that diabetes duration might play a role in mediating the relationship between C-peptide and corneal nerve parameters. Finally, we observed that fasting C-peptide levels declined with duration of diabetes. All these data suggested that fasting C-peptide levels might be both an indicator of beta-cell function and a marker of disease severity (such as diabetic corneal neuropathy) and duration.

Some limitations of this study should be noted. First, as this is a cross-sectional study, it is not possible to determine causality between fasting C-peptide levels and corneal nerve damage. Second, we only included Chinese with T2DM in this study and the results need to be confirmed in patients with other ethnicities and patients with T1DM. Third, we did not evaluate the presence of diabetic peripheral and autonomic neuropathy in the population as it was closely associated with the CCM alterations. The choice of a very sensitive testing (such as CCM) might seem to be undermined by the long disease duration of the study population and the lack of a comparison with standard testing modalities for diabetic polyneuropathy. Fourth, we did not exclude patients who were receiving insulin therapy as insulin administration might affect fasting C-peptide levels, especially the use of long-acting analogues. However, to exclude the effect of insulin therapy on results, insulin injections were stopped 24 hours before blood collection for patients on long-acting insulin treatment and we had adjusted for patients' antidiabetic medication (including insulin therapy) in models of Table 3. Lastly, some confounding factors (such as inflammatory factors) that might affect the levels of fasting C-peptide or DPN may not have been adjusted for. However, in order to reduce the influence of such factors as far as possible, we had a strict exclusion criterion as described in Methods.

In conclusion, we found that fasting C-peptide levels were positively associated with CNFL, CNFD, and CNBD and negatively correlated with duration of diabetes. Fasting C-peptide levels might be both an indicator of beta-cell function and a marker of disease severity (such as diabetic corneal neuropathy) and duration. Further prospective and intervention studies are now required to investigate the role of C-peptide in corneal neuropathy and T2DM DPN.

## Figures and Tables

**Table 1:** Characteristics of participants by fasting C-peptide quartiles.

Characteristics	Quartile 1 (*n* = 40)	Quartile 2 (*n* = 40)	Quartile 3 (*n* = 40)	Quartile 4 (*n* = 40)	*P* value^a^
Female (*n* (%))	21 (52.5%)	20 (50.0%)	14 (35.0%)	17 (42.5%)	0.387
Age (years)	56.73 ± 9.11	57.98 ± 7.12	56.78 ± 8.92	55.73 ± 9.86	0.727
BMI (kg/m^2^)	23.94 ± 3.78	25.94 ± 3.43^b^	26.65 ± 3.44^b^	27.77 ± 4.29^bc^	**<0.001**
Systolic BP (mmHg)	135.20 ± 21.04	142.25 ± 22.35	143.50 ± 17.19	141.30 ± 21.15	0.280
Diastolic BP (mmHg)	78.30 ± 13.47	80.23 ± 13.61	81.63 ± 11.94	82.13 ± 12.52	0.550
FBG (mmol/L)	8.22 ± 2.40	8.27 ± 2.06	8.09 ± 2.76	7.81 ± 2.25	0.824
HbA1c (%)	8.67 ± 1.88	8.14 ± 1.75	8.44 ± 1.83	7.71 ± 1.59^b^	0.092
Fasting C-peptide (ng/mL)	0.68 ± 0.23	1.23 ± 0.15^b^	1.75 ± 0.17^bc^	2.77 ± 0.73^bcd^	**<0.001**
Duration of diabetes (years)	12.00 (7.00-16.75)	12.50 (8.00-17.00)	10.00 (4.25-16.00)	7.00 (2.63-10.00)^bc^	**0.002**
Triglyceride (mmol/L)	1.43 (0.94-2.27)	1.41 (0.98-2.47)	1.31 (0.97-2.55)	1.37 (1.05-2.38)	0.906
TC (mmol/L)	5.24 ± 1.21	4.45 ± 1.26^b^	4.43 ± 0.97^b^	4.63 ± 1.11^b^	**0.006**
Creatinine (*μ*mol/L)	63.60 ± 14.11	63.30 ± 13.04	71.78 ± 33.05	63.41 ± 21.81	0.234
eGFR (mL/min/1.73 m^2^)	96.92 ± 13.67	97.35 ± 13.29	95.01 ± 20.15	102.04 ± 20.73	0.316
Insulin treatment (*n* (%))	34 (85.0%)	24 (60.0%)^b^	20 (50.0%)^b^	13 (32.5%)^bc^	**<0.001**
Long-acting insulin analogues (*n* (%))	31 (77.5%)	23 (57.5%)^b^	16 (40.0%)^b^	9 (22.5%)^bc^	**<0.001**
Metformin (*n* (%))	25 (62.5%)	33 (82.5%)	33 (82.5%)	29 (72.5%)	0.118
Alpha glucosidase inhibitor (*n* (%))	13 (32.5%)	16 (40.0%)	18 (45.0%)	23 (57.5%)^b^	**0.003**
Other antidiabetic medications (*n* (%))	3 (7.5%)	2 (5.0%)	10 (25.0%)^bc^	4 (10.0%)	**0.026**
CNFL (mm/mm^2^)	13.94 ± 4.16	13.38 ± 3.89	16.38 ± 2.98^bc^	16.39 ± 3.60^bc^	**<0.001**
CNFD (number/mm^2^)	20.58 ± 9.90	19.29 ± 7.03	23.60 ± 7.63^c^	24.16 ± 7.23^bc^	**0.019**
CNBD (number/mm^2^)	33.19 ± 26.06	28.01 ± 18.70	40.20 ± 18.42^c^	44.75 ± 22.58^bc^	**0.004**

The data are expressed as the means ± SD or median (interquartile range). BMI: body mass index; BP: blood pressure; FBG: fasting blood glucose; TC: total cholesterol; eGFR: estimated glomerular filtration rate; CNFL: corneal nerve fiber length; CNFD: corneal nerve fiber density; CNBD: corneal nerve branch density. ^a^Difference between four groups; ^b^*P* < 0.05 compared with Quartile 1 group; ^c^*P* < 0.05 compared with Quartile 2 group; ^d^*P* < 0.05 compared with Quartile 3 group.

**Table 2:** Partial correlation analysis of metabolic indexes with corneal nerve parameters (adjusted for age and gender).

Characteristics	CNFL		CNFD		CNBD	
	*r*	*P* value	*r*	*P* value	*r*	*P* value
BMI	0.128	0.108	0.067	0.405	0.033	0.689
Systolic BP	-0.040	0.614	-0.057	0.480	0.038	0.645
Diastolic BP	-0.009	0.911	-0.004	0.960	0.027	0.743
FBG	-0.153	0.055	-0.156	0.050	-0.114	0.161
HbA1c	-0.211	**0.008**	-0.117	0.142	-0.143	0.077
Duration of diabetes	-0.210	**0.008**	-0.107	0.181	-0.090	0.269
Triglyceride	0.045	0.575	0.038	0.640	0.036	0.661
TC	-0.167	**0.036**	-0.152	0.058	-0.153	0.060
Creatinine	-0.014	0.866	0.007	0.929	0.026	0.752
eGFR	-0.009	0.908	0.009	0.915	-0.051	0.535
Fasting C-peptide	0.245	**0.002**	0.180	**0.024**	0.214	**0.008**

**Table 3:** Multiple linear regression analysis of C-peptide with corneal nerve parameters (as dependent variable).

Models	Characteristics	CNFL		CNFD		CNBD	
		*β* coefficient (95% CI)	*P* value	*β* coefficient (95% CI)	*P* value	*β* coefficient (95% CI)	*P* value
Model 1	Fasting C-peptide (ng/mL)	1.160 (0.480 to 1.839)	**0.001**	1.855 (0.403 to 3.306)	**0.013**	5.776 (1.802 to 9.750)	**0.005**
Model 2	Fasting C-peptide (ng/mL)	0.828 (0.010 to 1.647)	**0.047**	1.227 (-0.573 to 3.027)	0.180	5.297 (0.302 to 10.291)	**0.038**
Model 3	Fasting C-peptide (ng/mL)	0.716 (-0.117 to 1.549)	0.091	1.158 (-0.685 to 3.000)	0.216	5.104 (-0.005 to 10.212)	0.050

Model 1: unadjusted. Model 2: adjusted for age, gender, BMI, SBP, HbA1c, triglyceride, TC, eGFR, insulin treatment, metformin, alpha glucosidase inhibitor, and other antidiabetic medications. Model 3: adjusted for age, gender, BMI, SBP, HbA1c, triglyceride, TC, eGFR, insulin treatment, metformin, alpha glucosidase inhibitor, other antidiabetic medications, and duration of diabetes.

## Data Availability

The data used to support the findings of this study are available from the corresponding author upon request.

## Supplementary Materials

Supplementary Materials Table 1: characteristics of all participants. Table 2: multiple linear regression analysis of diabetes duration with corneal nerve parameters (as dependent variable).
