# Supplementary material for: The Association of Fasting C-peptide with Corneal Neuropathy in Patients with Type 2 Diabetes
Source: J Diabetes Res. 2020 Dec 2;2020:8883736. doi: 10.1155/2020/8883736 (PMC7725581; doi:10.1155/2020/8883736)
Supplement: Supplementary Materials — Table 1: characteristics of all participants. Table 2: multiple linear regression analysis of diabetes duration with corneal nerve parameters (as dependent variable). [file 8883736.f1.docx]

**Supplementary material:**

**Table 1** Characteristics of all participants.

| Characteristics | All subjects |
| --- | --- |
| Female [n (%)] | 72 (45%) |
| Age (years) | 56.80±8.76 |
| BMI (kg/m^2^) | 26.07±3.97 |
| Systolic BP (mmHg) | 140.56±20.58 |
| Diastolic BP (mmHg) | 80.57±12.87 |
| FBG (mmol/L) | 8.10±2.37 |
| HbA1c (%) | 8.24±1.78 |
| Fasting C-peptide (ng/mL) | 0.68±0.23 |
| Duration of diabetes (years) | 10.00 (6.00-15.75) |
| Triglyceride (mmol/L) | 1.40 (0.98-2.399) |
| TC (mmol/L) | 4.69±1.18 |
| Creatinine (μmol/L) | 65.52±22.10 |
| eGFR (mL/min/1.73 m^2^) | 97.85±17.35 |
| Insulin treatment [n (%)] | 91 (56.9%) |
| Long-acting insulin analogues [n (%)] | 79 (49.4%) |
| Metformin [n (%)] | 120 (75.0%) |
| Alpha glucosidase inhibitor [n (%)] | 70 (43.8%) |
| Other antidiabetic medications [n (%)] | 19 (11.9%) |
| CNFL (mm/mm^2^) | 15.02±3.90 |
| CNFD (number/mm^2^) | 21.91±8.21 |
| CNBD (number/mm^2^) | 36.67±22.40 |
| CHD [n (%)] | 35 (21.9%) |
| Hypertension [n (%)] | 87 (54.4%) |
| Diabetic retinopathy [n (%)] | 83 (51.9%) |
| Diabetic kidney disease [n (%)]^a^ | 31 (29.2%) |

The data are expressed as the means ± SD or median (interquartile range). BMI, body mass index; BP, blood pressure; FBG, fasting blood glucose; TC, total cholesterol; eGFR, estimated glomerular filtration rate; CNFL, corneal nerve fiber length; CNFD, corneal nerve fiber density; CNBD, corneal nerve branch density; CHD, coronary heart disease. ^a^: 105 subjects can be diagnosed as diabetic kidney disease or not, because only 105 subjects tested urine albumin-creatinine ratio.

**Table 2** Multiple linear regression analysis of diabetes duration with corneal nerve parameters (as dependent variable).

|  |  | CNFL | | CNFD | | CNBD | |
| --- | --- | --- | --- | --- | --- | --- | --- |
| Models | Characteristics | β Coefficient | *P*-value | β Coefficient | *P*-value | β Coefficient | *P*-value |
| Model 1 | diabetes duration | -0.139 | **0.002** | -0.187 | 0.052 | -0.403 | 0.134 |
| Model 2 | diabetes duration | -0.108 | **0.027** | -0.104 | 0.324 | -0.247 | 0.402 |
| Model 3 | diabetes duration | -0.087 | 0.083 | -0.063 | 0.562 | -0.084 | 0.782 |

Model 1: Unadjusted.

Model 2: Adjusted for age, gender, BMI, SBP, HbA1c, triglyceride, TC, eGFR.

Model 3: Adjusted for age, gender, BMI, SBP, HbA1c, triglyceride, TC, eGFR and C-peptide.
